# Chronic Inhibition, Self-Control and Eating Behavior: Test of a ‘Resource Depletion’ Model

**DOI:** 10.1371/journal.pone.0076888

**Published:** 2013-10-17

**Authors:** Martin S. Hagger, Giulia Panetta, Chung-Ming Leung, Ging Ging Wong, John C. K. Wang, Derwin K. C. Chan, David A. Keatley, Nikos L. D. Chatzisarantis

**Affiliations:** 1 Health Psychology and Behavioural Medicine Research Group, School of Psychology and Speech Pathology, Curtin University, Perth, Western Australia, Australia; 2 School of Psychology, University of Nottingham, Nottingham, Nottinghamshire, United Kingdom; 3 National Institute of Education, Singapore, Singapore; University of Udine, Italy

## Abstract

The current research tested the hypothesis that individuals engaged in long-term efforts to limit food intake (e.g., individuals with high eating restraint) would have reduced capacity to regulate eating when self-control resources are limited. In the current research, body mass index (BMI) was used as a proxy for eating restraint based on the assumption that individuals with high BMI would have elevated levels of chronic eating restraint. A preliminary study (Study 1) aimed to provide evidence for the assumed relationship between eating restraint and BMI. Participants (N = 72) categorized into high or normal-range BMI groups completed the eating restraint scale. Consistent with the hypothesis, results revealed significantly higher scores on the weight fluctuation and concern for dieting subscales of the restraint scale among participants in the high BMI group compared to the normal-range BMI group. The main study (Study 2) aimed to test the hypothesized interactive effect of BMI and diminished self-control resources on eating behavior. Participants (N = 83) classified as having high or normal-range BMI were randomly allocated to receive a challenging counting task that depleted self-control resources (ego-depletion condition) or a non-depleting control task (no depletion condition). Participants then engaged in a second task in which required tasting and rating tempting cookies and candies. Amount of food consumed during the taste-and-rate task constituted the behavioral dependent measure. Regression analyses revealed a significant interaction effect of these variables on amount of food eaten in the taste-and-rate task. Individuals with high BMI had reduced capacity to regulate eating under conditions of self-control resource depletion as predicted. The interactive effects of BMI and self-control resource depletion on eating behavior were independent of trait self-control. Results extend knowledge of the role of self-control in regulating eating behavior and provide support for a limited-resource model of self-control.

## Introduction

Research from numerous theoretical perspectives has implicated self-control in the regulation of health-related behavior [1], [2]. Low levels of self-control have been associated with reduced compliance with behaviors such as physical activity [3], [4], alcohol [5] and smoking [6] cessation, and following a healthy diet [4], [7], which is likely to have long term detrimental effects on salient health-related outcomes including risk of chronic disease [1]. Although self-control has been shown to be positively related to healthy eating patterns and successful weight control [8], [9], [10], few studies have examined the role that self-control plays in managing eating behavior in chronic dieters and individuals with problems with eating restraint, such as those with high BMI [11]. The current research aims to examine the effect of self-control on eating behavior in individuals with high BMI from the perspective of the strength model, a topical theory of self-control.

The ‘strength’ model of self-control is a relatively recently-developed theoretical approach that has been adopted to understand the effects of self-control on health behavior [1], [12], [13]. In this model, self-control is conceptualized as a limited resource that governs individuals’ capacity for self-control. According to the model, self-control resources become depleted through engaging in tasks or activities that demand self-control and, without sufficient rest or recovery, performance on subsequent activities requiring self-control is likely to be impaired. Baumeister et al. [12] likened self-control to a kind of strength or energy; just as a muscle becomes tired after a period of exertion so self-control resources become exhausted when they are consumed through engagement in self-control tasks. The state of reduced self-control capacity has been termed *ego-depletion*.

The strength model has ramifications for behavior in numerous everyday contexts in which self-control is important, including health-related behavior. People experience demands on their self-control resources on a daily basis. Activities such as persisting with extended boring tasks like filing or typing, dealing with difficult customers, coping with setbacks when under time pressure such as meeting deadlines, making informed choices between consumer products, and resisting temptations to eat in excess, smoke cigarettes, or drink too much alcohol, particularly if one is trying to cut down or quit, all require people to resist temptations or persist when the ‘path of least resistance’ is to desist. Such tasks are likely to diminish self-control resources in the short term and, without sufficient rest or recovery, an individual is likely to have difficulty in performing subsequent activities that require self-control. For example, after a work day in which an office clerk is told by his boss to do an intensely boring filing task without a break, he may have reduced capacity to resist the temptation to drink more alcohol with his friends after work even though he is trying to cut down on his intake.

Evidence in support of the strength model of self-control has largely come from experimental research using a method known as the dual-task procedure [14]. In the procedure, participants engage in two consecutive laboratory-based tasks. Experimental-group participants engage in an initial task requiring self control while control-group participants engage in a similar task that does not require self-control. Both groups then engage in a second task that requires self-control in a different domain to the first. The extent to which the second-task performance of participants’ assigned to the experimental group is impaired relative to participants allocated to the control group provides confirmation of the ego-depletion effect. Research adopting the dual-task procedure has provided considerable support for the ego-depletion effect across numerous domains of self-control including tasks that require impulse, emotion, attention, and thought control [15].

Much of the research on the strength model has focused on a *state* perspective and examined how short-term depletion of self-control resources results in reduced capacity for subsequent tasks requiring self-control. However, the model also encompasses a trait perspective which posits that individuals also differ in their self-control capacity [8], [16]. Trait self control is viewed as the extent of an individual’s self-control reserve or capacity [8], [16]. Individuals with high levels of trait self-control would have more of the resource at their disposal and, therefore, be less susceptible to the deleterious effects of ego-depletion on task performance. This implies that trait self-control serves to moderate of the effects of ego-depletion on subsequent task performance and there is some evidence to support this effect [17], [18].

Researchers have also hypothesized that long-term demands on self-control may impair self-control capacity [19], [20]. For example, dieters are frequently faced with situations in which they must exert self-control over their eating behavior (i.e., resisting tempting foods). This may lead to a chronic reduction in self-control capacity and a vulnerability to ego-depletion should they engage in subsequent tasks or activities that place demands on an individual’s self-control resources. In the context of dieting, Vohs and Heatherton [19] examined the proposed effects of chronic self-control resource depletion on individuals’ self-control capacity. They identified dieters as a group of individuals who engaged in long-term impulse suppression through their avoidance of tempting foods. Dieters whose state levels of self-control were depleted using an emotion suppression task and were exposed to highly tempting foods consumed more ice-cream in a taste-and-rate task relative to non-depleted and low-temptation groups. The authors concluded that “the existence of chronic inhibitions, when combined with situational conditions requiring effortful self-regulation, can decrease ability to self-regulate” (p. 254).

Vohs and Heatherton [19] defined dieters as those with high levels of eating restraint and proposed that these individuals would exhibit a generalized, long-term tendency to restrict their eating behavior. These individuals, therefore, constituted a group with chronic dietary inhibitions. The long term inhibition of eating behavior left dieters with diminished self-control resources and vulnerable to state self-control resource depletion and subsequent self-regulatory failure when presented with tempting foods. However, Vohs and Heatherton did not link levels of eating restraint to clinically-significant indicators of health and increased risk of chronic disease, such as BMI. BMI may serve as an indicator of eating restraint as individuals with high BMI are likely to regularly experience the need to restrain their eating behavior in the face of the many temptations that likely trigger a salient and potent desire to eat. Indeed, BMI has been linked to increased impulsiveness when it comes to eating [21] as well as elevated desire for eating restraint [22], [23].

### The Present Study

In the current research we aimed to extend Vohs and Heatherton’s findings by examining the interactive effects of BMI and state ego-depletion on food consumed in a taste-and-rate task. We considered BMI an indicator of eating restraint. Previous research has shown a significant relationship between eating restraint and BMI with large effect sizes in student and community samples [22], [23]. BMI is also clinically relevant as it reflects groups at increased risk of certain chronic illnesses such as cardiovascular disease, hypertension, and hypercholesterolemia [24]. We therefore proposed that individuals classified as high BMI would be more likely to engage in chronic restraint of eating behavior, and therefore long-term exertion of self-control, compared to low BMI participants.

In Study 1, we aimed to provide initial evidence to confirm the significant association between eating restraint, as measured by the Restraint Scale (RS) [25], and BMI. We expected that individuals with high BMI, computed as weight (kilograms) divided by height (meters) squared, would score significantly higher on the subscales of the RS relative to individuals with BMI in the normal range. In Study 2, following Vohs and Heatherton, we aimed to test our main hypothesis that the long-term exertion of self-control among individuals with high BMI would leave them vulnerable to overeat when their self-control resources were depleted. Using a dual-task experimental procedure, we set out to manipulate the self-control resources of study participants classified as high and normal-range BMI and examine their eating behavior in an ostensible taste-and-rate task for tempting foods. We predicted that high-BMI participants would eat more food in the taste-and-rate task relative to normal-range BMI participants but only under when self-control resources were depleted. The hypothesized interaction effect is similar to that found by Vohs and Heatherton [19] for the effect of eating restraint and ego-depletion on eating behavior. In addition, we hypothesized that the effect of BMI on eating behavior would be a function of both state availability of self-control resources and trait levels of self-control. This is because trait-self-control may indicate individuals’ overall capacity for self-regulation and may therefore moderate the hypothesized interactive effect of BMI and self-control resource depletion on eating behavior. We therefore aimed to include self-control as an additional independent variable in our analysis.

## Study 1

A key assumption in the current research was that BMI served as an indicator of people engaged in the chronic inhibition of their eating behavior. Although previous research has demonstrated associations between eating restraint and BMI, we sought to provide independent evidence to corroborate these findings [22], [23]. The purpose of Study 1 was, therefore, to provide preliminary support for our contention that individuals with high BMI are likely to have elevated eating restraint relative individuals with BMI in the normal range.

### Materials and Methods

#### Ethics statement

The research protocol was reviewed and approved by the Health Research Ethics Committee or Curtin University, Australia prior to data collection. Written informed consent was obtained from all participants.

#### Participants

We recruited a sample of undergraduate and postgraduate students and staff from Curtin University (N = 72, 32 males, 40 females; *M* age = 27.08, *SD* = 10.83). We recruited in exactly equal numbers of participants classified as high (BMI ≥25; n = 36, 17 males, 19 females; BMI *M* = 30.61, *SD* = 5.51) and normal-range (BMI<25; n = 36, 15 males, 21 females; BMI *M* = 21.42, *SD* = 1.50) BMI. Participants were students and staff who volunteered to participate in the study in response to a general email circular and posters distributed about campus.

#### Design and procedure

Participants were informed that they were participating in a brief survey on eating beliefs and perceptions. Participants were initially required to complete an online screening questionnaire comprising self-report measures of weight, height, gender, and age. Equal numbers of participants classified into high and normal-range BMI categories according to the screening questionnaire were asked to complete the 10-item Restraint Scale (RS) [25]. The inventory comprises two subscales: Weight Fluctuation (RS-WF) comprising 5 items (e.g., “In a typical week how much does your diet fluctuate?”) with responses made on 4-point scales anchored by 1 (*0–0.5 kg*) and 5 (*2.5 kg or greater*) and Concern for Dieting (RS-CD) comprising 5 items (e.g., “How often do you diet?”) with responses made on 4-point scales anchored by 1 (*never*) and 5 (*always*). After completing the questionnaire participants were debriefed and dismissed.

### Results

RS subscale scores were significantly higher in the high BMI group for the RS-WF (high BMI group, *M* = 2.88, *SD* = 0.51; normal-range BMI group, *M* = 1.97, *SD* = 0.60; *t*(70) = 6.99, *p*<.001, *d* = 1.67, *r*(72) = .68, *p*<.001) and the RS-CD (high BMI group, *M* = 2.71, *SD* = 0.50; normal-range BMI group, *M* = 2.22, *SD* = 0.49; *t*(70) = 4.23, *p* = .003, *d* = 1.01, *r*(72) = .52, *p*<.001) subscales compared to the low BMI group. Results suggest that individuals with high BMI report considerably higher restraint scores that those with normal-range BMI and corroborate previous research identifying differences among normal-weight and obese participants [22].

## Study 2

Based on the findings of Study 1, we assumed that participants with high BMI have higher levels of eating restraint and frequently engage in behaviors to reduce their food intake. We proposed that although high BMI individuals have higher restraint, the chronic nature of their restraint over eating is likely to lead them to be vulnerable to acute depletion of self-control resources. In Study 2 we proposed to test our main hypothesis that individuals with high BMI would be more vulnerable to short term depletion of self-control resources and would be more likely to eat more in an ostensible taste-and-rate task of tempting foods when depleted relative to individuals with lower BMI. We therefore expected a significant interaction effect of self-control resource depletion and BMI on food consumed in the taste-and-rate task.

### Materials and Methods

#### Ethics statement

The research protocol was reviewed and approved by the Research Ethics Committee of the School of Psychology, University of Nottingham, UK prior to data collection. Written informed consent was obtained from all participants.

#### Participants

Participants were undergraduate psychology students (N = 83, 38 males, 45 females; *M* age = 19.00, *SD* = 1.21) who volunteered to participate in the study in exchange for course credit. We recruited in approximately equal numbers of participants classified as high (BMI ≥25; n = 25, 12 males, 13 females), mid-range (BMI = 21.00 to 24.99; n = 27, 12 males, 15 females), and low (BMI = 19.00 to 20.99; n = 31, 14 females, 17 males) BMI. Participants were recruited based on their self-reported height and weight ascertained from a preliminary health questionnaire administered to the entire cohort of psychology students earlier in the semester. Eligible participants were contacted by email and invited to participate in study ostensibly on cognitive abilities. Participants were blinded to the true purpose of the experiment until the study was complete to minimize the potential of participant expectation affecting responses. Participants were asked to refrain from eating two hours prior to their visit to the laboratory.

#### Design and procedure

A dual-task experimental paradigm was adopted to manipulate self-control resources. Participants were randomly allocated to experimental (ego-depletion) and control (no depletion) groups using an online research randomizer tool [26]. Participants were tested individually in a laboratory. Participants were invited into the laboratory by a female researcher and asked to perform an initial task introduced as a “test of mathematical ability” as part of an experiment on “cognitive abilities”. Participants assigned to the experimental condition were asked to count backwards from 1000 in multiples of seven while standing on one leg. Participants allocated to the control condition were also asked to count down from 1000 but were to do so in multiples of five whilst standing normally. This task is based on a test used to assess automatization ability in dyslexics and has been previously adopted as a means to evoke self-control resource depletion [27], [28]. The version of the task presented in the ego-depletion condition was expected to be more demanding of participants’ self-control resources as they were obliged to resist the temptation to quit due to the difficulty of simultaneously coordinating balance and engaging in the complex counting task. Performance on this version of the task was compared to the relatively benign version given to participants in the control condition. After completing the counting task participants completed questionnaire measures of task difficulty, effort, and fatigue, and positive and negative affect.

Participants were next asked to engage in a taste-and-rate task presented as a separate experiment. As a cover story, participants were informed that they were participating in some market research examining their perceptions of certain foods. They were asked to sit at a desk. On the desk were two plates of tempting food; one containing small equal-sized 5 g pieces of cookies and one containing Smarties™ (colored candy-coated chocolate). Participants were asked to taste the foods individually and rate them on several scales. The experimenter told participants they could eat as much of the foods as they wanted and could take as much time as they needed to make their ratings. In reality, all participants were limited to 10 minutes for each food and all took less than the allotted time to finish their ratings. The experimenter left the room telling participants to ring a buzzer when they had finished. The experimenter covertly observed participants during the taste-and-rate task using a concealed camera. After the experimenter had been summoned, participants were asked to complete measures of task perceptions and trait self-control. Participants were then informed that the experiment was over. A funnel debriefing procedure was used to probe participants for suspicion as to the purpose of the tasks and whether the counting task was related to the taste-and-rate task.

#### Measures

Positive and negative affect. Measures of positive and negative affect were included to ensure that the effects of self-control on eating behavior were not the result of an emotional response. Positive and negative affect was measured using the Brief Mood Introspection Scale (BMIS) [29]. Participants were presented with an initial common question “How do you feel right now?” followed by seven items tapping positive affect (e.g., “lively”, “peppy”, “happy”; α = .73) and nine items tapping negative affect (e.g., “drowsy”, “tired”, “gloomy”; α = .65). Responses were made on four-point scales ranging from 1 (*definitely do not feel*) to 4 (*definitely feel*).

#### Task perceptions

As a check that the manipulation of ego-depletion was successful, participants’ rated the counting task using single-item measures of difficulty, fatigue, and effort on scales ranging from 1 (*not at all*) to 7 (*very much*).

#### Trait self-control

We used the brief version of Tangney et al.’s [8] scale to measure individual differences in self-control capacity. Participants were asked to rate their personal disposition on 13 items (e.g., “I am good at resisting temptation”, I refuse things that are bad for me”, “People would say that I have an iron self-discipline”; α = .86) with responses made on five-point scales ranging from 1 (*not at all*) to 5 (*very much*).

#### Eating behavior

The primary dependent variable was the objective amount (pieces) of the two foods eaten in the course of the taste-and-rate task. This was ascertained by direct observation of the amount of each food participants ate during the task via the concealed camera. The observed amount was subsequently corroborated by a count of the amount of food remaining on the plates after the participant had left. Participants were also asked to self-report the amount of each food they had tasted.

### Results

#### Preliminary analyses

Correlations among study constructs are presented in Table 1. The ego-depletion experimental condition was computed as a dichotomous dummy-coded variable (0 = ego-depletion condition, 1 = control). Consistent with our use of multiple regression in subsequent analyses, we used correlations to test our manipulation checks and alternative hypotheses (e.g., emotional responses, taste perceptions) rather than *t*-tests. Conducting the analyses using *t*-tests revealed identical findings as the tests are statistically equivalent. Ego-depletion was significantly correlated with participants’ subjective ratings of difficulty, fatigue, and effort. Specifically, participants allocated to the ego-depletion condition rated the counting task as more difficult (ego-depletion condition, *M* = 5.50, *SD* = 0.99; control condition, *M* = 2.86, *SD* = 1.26; *r*(83) = .76, *p*<.001), fatiguing (ego-depletion condition, *M* = 4.90, *SD* = 1.06; control condition, *M* = 2.88, *SD* = 1.47; *r*(83) = .62, *p*<.001), and effortful (ego-depletion condition, *M* = 5.60, *SD* = 0.87; control condition, *M* = 3.84, *SD* = 1.59; *r*(83) = .57, *p*<.001) relative to participants assigned to the control condition. These items served as a manipulation check for the ego-depletion manipulation and indicated that the counting task successfully evoked an ego-depleted state among participants allocated to the ego-depletion condition. Ego depletion was also significantly correlated with observed amount of cookies (ego-depletion condition, *M* = 1.98, *SD* = 1.51; control condition, *M* = 1.57, *SD* = 0.82; *r*(83) = .41, *p*<.001) and Smarties™ (ego-depletion condition, *M* = 5.60, *SD* = 4.31; control condition, *M* = 3.72, *SD* = 2.85; *r*(83) = .49, *p*<.001) eaten in the taste-and-rate task. BMI was significantly and negatively correlated with trait self-control (*r*(83) = −.32, *p*<.001). Neither ego-depletion nor BMI were significantly correlated with negative and positive affect indicating that the effects of ego-depletion and BMI on eating behavior were independent of emotional responses to eating. Although trait self-control was significantly correlated with BMI, correlations of this variable with amount of cookies or Smarties™ eaten failed to reach significance. It was therefore unlikely that trait self-control would act as a moderator the proposed effects. Nevertheless, we included trait self-control as a covariate in all analyses to ensure that hypothesized effects were unaffected by variations in trait self-control.

**Table 1:** Intercorrelations Among Study Variables.

Variable	1	2	3	4	5	6	7	8	9
1. Ego-depletion^a^	–								
2. BMI	.03	–							
3. Difficulty	.76***	.08	–						
4. Fatigue	.62***	.13	.70***	–					
5. Effort	.57***	.16	.77***	.52***	–				
6. Positive affect	−.07	−.18	−.09	−.15	−.15	–			
7. Negative affect	.14	.01	.22*	.24*	.22*	−.38***	–		
8. Amount eaten (cookies)	.41***	.21	.26*	.35***	.16	−.10	.03	–	
9. Amount eaten (Smarties™)	.49***	.25*	.43***	.33***	.31***	−.12	.16	.54**	–
10. Trait self-control	−.07	−.32**	.02	−.14	−.04	.25*	−.06	−.19	−.21

*Note.* ^a^Dichotomous dummy-coded variable representing ego-depletion experimental condition (1 = ego-depletion, 0 = control).

* *p*<.05,

** *p*<.01,

** *p*<.001.

#### Main analyses

**Figure 1:**
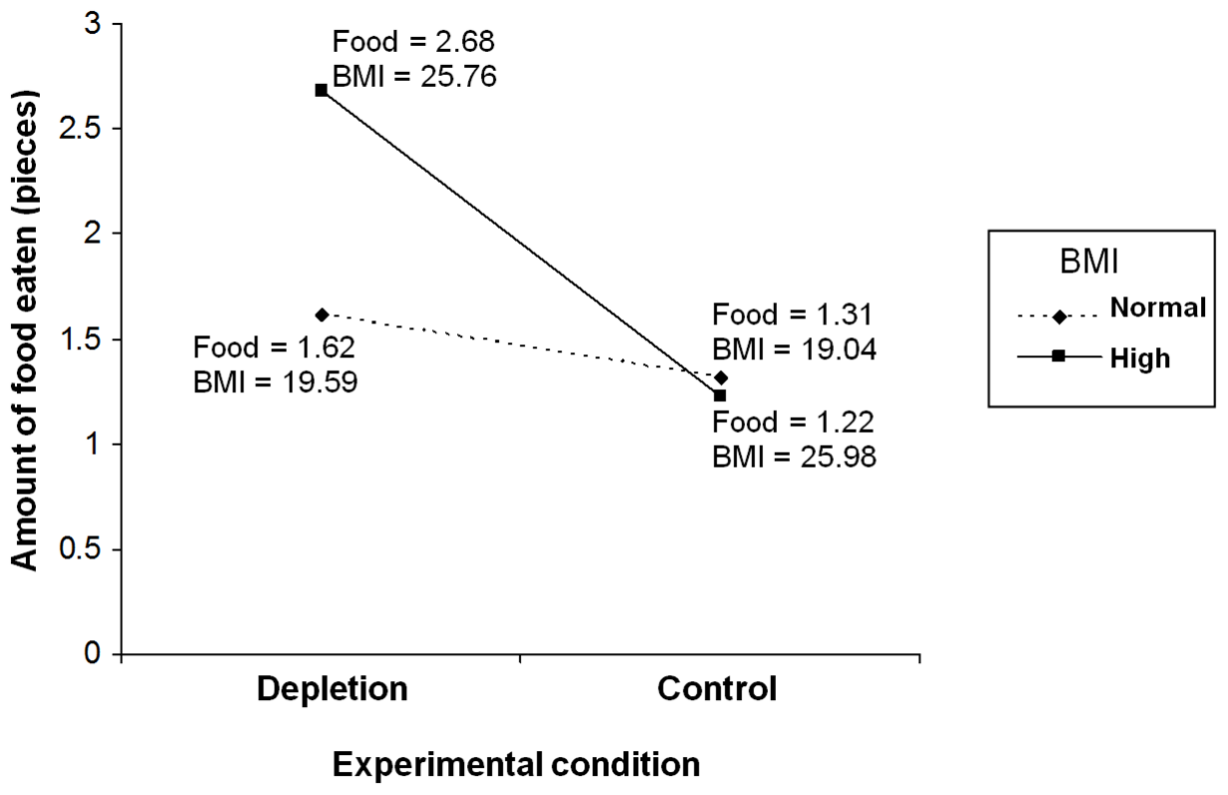
Simple slopes analysis of amount of food eaten (cookies) in taste-and-rate task as a function of ego-depletion condition and BMI. *Note*. Food = Amount of food eaten; BMI = Body Mass Index.

**Figure 2:**
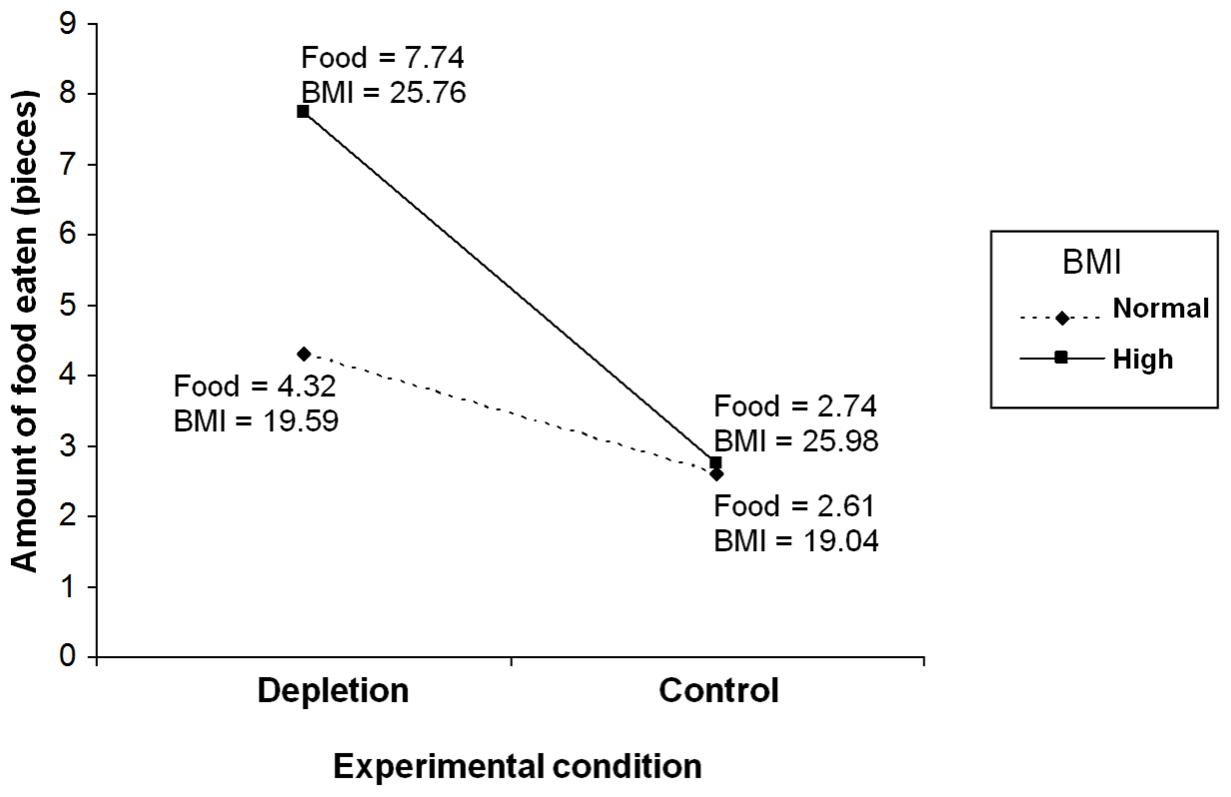
Simple slopes analysis of amount of food eaten (Smarties™) in taste-and-rate task as a function of ego-depletion condition and BMI. *Note*. Food = Amount of food eaten; BMI = Body Mass Index.

Given that BMI was a continuous independent variable and the fact that we had unequal numbers of participants in the high and normal-range BMI groups, we opted to test the hypothesized interactive effect of ego-depletion and BMI on amount of food eaten in the taste and rate task using multiple linear regression with tests for interaction (moderator) effects following Aiken and West’s [30] methods. Specifically, amount of food eaten in the taste-and-rate task was simultaneously regressed on ego-depletion condition (as a dichotomous dummy-coded variable), BMI (as a mean-centered continuous variable), and the multiplicative composite of these variables representing their interaction. Two separate analyses were conducted with amount of cookies and Smarties™ eaten as dependent variables, respectively. Mean-centered trait self-control was also included as an independent predictor variable in each analysis in a separate step. Results of the regression analyses are provided in Table 2. For each analysis there were significant main effects for ego-depletion and BMI, and a significant ego-depletion×BMI interaction effect, on amount of food eaten. There was no significant effect for trait self-control in either analysis. We probed the interactions using simple slopes analyses. In each analysis, we calculated separate slopes for the effect of ego-depletion on food eaten at two standard deviations above and below the mean for BMI. Results of the analyses are presented in Figures 1 and 2. For participants with lower BMI, there was no difference in the amount of cookies (β = −0.02, *SE* = .23, *t*(88) = −0.09, *p* = .927) and Smarties™ (β = 0.10, *SE* = .27, *t*(88) = 0.38, *p* = .705) eaten for participants allocated to the ego-depletion and control conditions. However, among participants with higher BMI, participants assigned to the ego-depletion condition consumed significantly more cookies (β = .42, *SE* = .12, *t*(79) = 3.55, *p*<.001) and Smarties™ (β = .42, *SE* = .11, *t*(79) = 3.80, *p*<.001) than participants assigned to the control groups (cookies, β = −.07, *SE* = .23, *t*(79) = −.31, *p* = .760; Smarties™, β = .04, *SE* = .28, *t*(79) = .15, *p*.879).

**Table 2:** Results of Moderated Multiple Linear Regression Analyses.

Independent variable	Dependent variable							
	Amount of cookies eaten^a^				Amount of Smarties™ eaten^b^			
	*B*	*SE*	*t*	*p*	*B*	*SE*	*t*	*p*
Ego-depletion^c^	−0.74	0.21	3.49	<.001	2.93	0.63	4.61	<.001
BMI	−0.06	0.15	−0.36	.721	0.38	0.01	0.08	.934
Ego−depletion × BMI	0.65	0.24	2.72	.008	1.86	0.72	2.60	.011
Trait self-control	−0.05	0.11	−0.46	.659	−0.19	0.33	−0.57	.571

*Note*. BMI = Body mass index.

a R^2^ = .25, *F*(4,78) = 6.86, *p*<.001;

b R^2^ = .36, *F*(4,78) = 10.99, *p*<.001;

c Dummy-coded variable representing ego-depletion experimental condition (0 = ego-depletion, 1 = control).

As a BMI greater than 25 represents an ‘overweight’ classification by international consensus and is associated with significantly higher risk of chronic disease [31], we thought it prudent to conduct an analysis isolating participants classified as high BMI from those with normal-range BMI to ensure that the effects found in the regression held for participants classified into the clinically-relevant categories of BMI. As a consequence, we conducted two focused-contrast ANOVAs to test the hypothesis that the amount of food eaten in the ostensible taste-and-rate task was significantly higher among high BMI participants under depletion compared to normal-range BMI participants regardless of depletion condition and high BMI participants allocated to the no-depletion condition. We classified participants into high (BMI ≥25; n = 25) and normal-range (BMI<25; n = 58) BMI categories. In the analyses, high BMI participants assigned to the depletion condition were allocated a weight of -3 while participants assigned to the no-depletion condition and low BMI participants assigned to the depletion condition were each allocated a weight of +1 according to Rosenthal and Rosnow’s [32] recommendations. Trait self-control was also included as a covariate. Dependent variables were amount of cookies and Smarties™ eaten, respectively, for each of the analyses. The analyses revealed a significant contrast effect with high BMI participants in the depletion condition consuming significantly more cookies (*M* = 2.85, *SD* = 1.57; *F*(3, 78) = 8.82, *p*<.001, η^2^_p_ = .25) and Smarties™ (*M* = 8.15, *SD* = 4.52; *F*(3, 78) = 11.68, *p*<.001, η^2^_p_ = .31) relative to the other groups (cookies, *M* = 1.48, *SD* = 0.82; Smarties™, *M* = 3.57, *SD* = 2.67). There was no effect for trait self-control in either analysis (cookies, *F*(1, 78) = 0.71, *p* = .40, η^2^_p_ = .01; Smarties™, *F*(1, 78) = 1.04, *p* = .31, η^2^_p_ = .01).

## Discussion

The purpose of the present research was to test whether individuals with high BMI, who are likely to be engaged in the chronic restraint of their eating behavior through dieting, tended to have difficulty in moderating their eating behavior when self-control resources were depleted. In our initial study (Study 1), we aimed to corroborate the assumption that individuals high in BMI would score highly on measures of eating restraint. Consistent with our predictions, results revealed that individuals classified as high BMI scored significantly higher on the subscales of the restraint scale relative to those with BMI falling within the normal range. In our main study (Study 2) we examined the effects of manipulating self-control resources on food eaten in an ostensible taste-and-rate task in participants classified into high and normal-range BMI categories. We found that participants with high BMI that engaged in a task that depleted their self-control resources consumed significantly more food compared to high BMI participants that did not engage in a depleting task and normal-range BMI participants regardless of depletion status. The effects of BMI and self-control resource depletion on eating behavior were independent of individual differences in trait self-control.

Results of the main study suggest that individuals with high BMI, who likely engage in frequent attempts to restrain their eating, to experience difficulties in regulating their eating when presented with tempting foods if their self-control resources are depleted. Consistent with the strength model of self-control, individuals engaged in repeated attempts at inhibition are likely to have reduced overall self-control capacity and further draws on their limited self-control resources may leave them vulnerable to subsequent temptations. Our results corroborate and extend Vohs and Heatherton’s [19] finding that dieters, classified on the basis of their eating restraint, were less likely to resist foods when their self-control resources had been depleted if they had been previously exposed to tempting foods. Importantly, our research used BMI as a proxy for chronic inhibition, based on evidence from previous research [22], [23] and the correlations between BMI and eating restraint found in Study 1. As individuals with a BMI greater than 25 have been shown to be at increased risk for a number of chronic illnesses, the present findings link the effects of reduced self-control and eating restraint on eating behavior with a known risk factor for chronic disease. Taken together, these findings seem to indicate that high BMI individuals, who are likely engaged in resisting temptations eating long-term, may have reduced self-control resource availability such that short-term self-control tasks are sufficient to undermine their resolve when presented with tempting foods.

It is important to note that the amount of food eaten in the taste-and-rate task by participants with high BMI that were not exposed to the situational depletion of self-control was not significantly different to the amount of food consumed by participants with normal-range BMI, regardless of whether the latter had had their situational self-control resources depleted. High BMI participants, therefore, still had sufficient self-control resources to regulate their eating behavior, provided their self-control resources were not depleted. Analogously, among participants with BMI in the normal range, those receiving a depleting task did not eat significantly more food in the taste-and-rate task than those that received a non-depleting task. This is probably because depleted participants in this group had sufficient self-control resources to resist temptation to consume more food because they did not have any long-term exposure to inhibition attempts.

We also found that the effect of chronic inhibition of eating on subsequent eating behavior was independent of trait self-control. Theoretically, it was expected that individuals with dispositionally lower capacity for self-regulation would likely have increased difficulty in resisting temptations and impulses, particularly if they had been involved in the chronic restraint of eating behavior (e.g., high-BMI participants) or were presented with situations that posed heightened demands on self-control resources (e.g., participants exposed to the ego-depletion experimental manipulation). According to the strength model, trait self-control is likely to interact with situational depletion of self-control resources, such that individuals with low trait self-control may be more susceptible to depletion [16], [33]. In the present study, we expected trait self-control to moderate the effect of eating restraint, as indicated by BMI, on eating behavior under conditions of low self-control resources. Our null finding for this effect may be an indication of the insensitivity of the trait self-control measure in tapping the essence of individual differences in restraint and impulsiveness. Individual differences in self-control likely encompasses a number of responses and processes involved in behavioral regulation such as resisting impulses, controlling attention, cognition, and emotion, and breaking habits and dominant responses, and a single trait measure such as the Tangney et al. [8] measure may not capture all these aspects. Recent research has adopted more ‘objective’ measures of dispositional self-control such as measures of response inhibition and executive functioning [34], [35]. Such measures may represent more robust means to assess trait self-control and may also capture aspects of the construct that have been hitherto unaccounted for in trait measures. Investigating whether these alternative measures serve to moderate the effects of chronic inhibition of eating and self-control resource depletion on eating behavior would be an important avenue for future research.

### Implications for Practice

The present study has a number of implications for practice that may enable high BMI individuals to better manage their eating behavior when exposed to tempting foods. First, managing demands on self-control resources may ensure that high BMI individuals are less likely to experience situations in which their self-control strength is compromised leaving them open to the tendency to overeat. For example, office workers should be provided with sufficient breaks when given cognitively demanding or tedious tasks. Second, reducing the frequency with which high BMI individuals engage in dieting, and, therefore, long-term inhibition of eating, may minimise the likelihood that short-term demands on self-control will lead to overeating. One way to do this would be to encourage high-BMI individuals to follow more consistent, healthy eating patterns rather than engaging in excessively-restrictive dieting. Finally, interventions to bolster self-control resources may assist in improving the availability of self-control resources in high BMI people and help them resist temptations to overeat when they arise. Evidence suggests that engaging in successive, discrete self-control tasks over time can lead to increases in self-control strength [36], [37], [38], [39]. It may, therefore, be possible to use such strategies to increase self-control strength among individuals with chronic inhibitions, like dieters, to reduce their vulnerability to temptations in the face of reduced self-control resources.

### Strengths, Limitations, and Conclusions

The present study had a number of strengths. The experimental design is advantageous as it enabled us to carefully control our independent manipulation of self-control and adopt an objective measure of behavior in the ostensible taste-and-rate task. In addition, we included BMI as an independent variable; a robust, objective means based on norms to identify individuals with higher and lower chronic inhibition of eating behavior. The use of BMI as an indicator of chronic inhibition and restraint is important as it links these variables with increased risk of chronic disease. We also adopted a contemporary theoretical approach to self-control in the strength model. Our findings extend the premises of this model by demonstrating that individuals engaged in chronic attempts at behavioral regulation were vulnerable to temptation when self-control resources were depleted and that these effects were independent of individual differences in trait self-control.

It would be remiss, however, not to acknowledge some potential limitations. First, we did not include a concurrent measure of eating restraint to corroborate our identification of participants as high and low chronic inhibitors according to BMI. We are confident that the classification successfully identified individuals with high and low restraint given the consistent evidence in the literature that BMI is highly correlated with restraint [22], [23]. Moreover, we provided important support for this relationship in Study 1 demonstrating that overweight and obese individuals with BMI greater than 25 scored significantly higher on restraint scales. However, it would have been desirable to have included a concurrent measure of eating restraint in the present study and should be acknowledged as a limitation. The inclusion of such a measure would not only have corroborated our assumed link between BMI and eating restraint, but it may also have provided the opportunity to test a potential mechanism for the proposed effects through mediation analysis. Specifically, testing whether eating restraint served to mediate the interactive effect of BMI and depletion on eating behavior would have elucidated whether our effects could be attributed to a chronic tendency to restrain eating among individuals with high BMI. We look to future research to investigate this possibility. Second, we also did not include a measure of dieting status. This would have provided further converging evidence that those identified as high and low on inhibition according to BMI were also more likely to be dieters.

In conclusion, the present study extends knowledge by demonstrating that high BMI individuals, who are likely to engage in chronic inhibition of their eating behavior through dieting, are more likely to overeat when their self-control resources are depleted. We also demonstrated that these effects are independent of trait self-control. Future research should examine the effects of intervention strategies to allay self-control depletion and reduce chronic inhibition in high BMI individuals such as promoting better recovery after everyday activities that require self-control, eating a more consistent, healthy diet, and improving self-control through training.
